# AMPK Suppresses Multiple Forms of Cell Death Including Disulfidptosis in Tumor-Associated Macrophages During Tumor Progression

**DOI:** 10.3390/ijms27146154

**Published:** 2026-07-09

**Authors:** Ruixuan Wang, Huan Wang, Dianyuan Zhao, Wenting Yang, Di Liu, Li Tang

**Affiliations:** State Key Laboratory of Medical Proteomics, National Center for Protein Sciences (Beijing), Academy of Military Medical Sciences, Beijing 102206, China

**Keywords:** TAMs, cell death, disulfidptosis, AMPK

## Abstract

Tumor-associated macrophages (TAMs) represent a predominant immune cell population within the tumor microenvironment (TME). To adapt to the metabolically hostile conditions of the TME, characterized by nutrient deprivation and accumulation of metabolic waste products, TAMs undergo metabolic reprogramming to evade cell death. These adaptations enable TAMs to utilize alternative metabolites as energy sources and mitigate metabolic stress through enhanced cystine uptake and activation of hypoxia-inducible factor pathways, thereby supporting their survival and function. However, the key molecular regulators that prevent TAMs death in response to dynamic metabolic changes during tumor progression remain poorly understood. Through integrated multi-omics analyses and experimental validation, we observed that increased AMPK activation during tumor progression is associated with transcriptomic and proteomic features indicative of reduced susceptibility of TAMs to multiple forms of cell death. Conditional deletion of AMPK in TAMs reprogrammed the expression of cell death-related genes and was associated with increased apoptosis, ferroptosis, and notably, disulfidptosis. Clinical correlation analyses revealed that AMPK activity in TAMs was inversely associated with the expression of disulfidptosis-, ferroptosis-, and apoptosis-related gene signatures. Furthermore, tumors characterized by concurrent enrichment of AMPK signaling and TAMs infiltration exhibited lower disulfidptosis, ferroptosis, and apoptosis signature scores, which were associated with a more malignant phenotype. Collectively, our findings suggest that AMPK activity is associated with TAM survival and tumor progression and with reduced susceptibility to multiple forms of cell death, including disulfidptosis. These findings provide evidence linking AMPK activity to metabolic adaptation and cell death resistance in TAMs and suggest its potential as a therapeutic target for cancer intervention.

## 1. Introduction

The tumor microenvironment (TME) is a metabolically dynamic system characterized by distinct features such as hypoxia, nutrient deprivation (e.g., glucose, amino acids, and glutamine), and the accumulation of metabolic byproducts, including lactate, adenosine, fatty acids, cholesterol, and reactive oxygen species [1,2]. These conditions drive intense competition for limited resources among cells within the TME, forcing them to undergo metabolic reprogramming to sustain their survival and function [1,3,4,5]. Cells that fail to adapt to these harsh metabolic conditions are prone to various forms of cell death resulting from imbalances in nutrient availability and metal homeostasis. Several metabolic stress-associated cell death modalities have been identified, including disulfidptosis, ferroptosis, cuproptosis, lysosome-dependent necrosis, alkaliptosis, and ammonia-induced cell death [6,7,8,9,10,11,12]. Accordingly, tumor-resident cells often develop reduced susceptibility to these cell death pathways to ensure survival in the hostile microenvironment.

Disulfidptosis, a newly identified and mechanistically distinct form of regulated cell death initially discovered in the TME, has recently emerged as an important focus of cancer research [9,11,13]. This form of cell death is characterized by unique morphological features, including cell shrinkage and F-actin contraction [9]. To counteract oxidative stress within the TME, tumor cells enhance cystine uptake through the cystine transporter solute carrier family 7 member 11 (SLC7A11) [13,14]. Intracellular cystine serves as a key precursor for glutathione (GSH) synthesis, an essential cofactor required for glutathione peroxidase 4 (GPX4)-mediated suppression of ferroptosis [15,16]. Under conditions of NADPH depletion, cells with high SLC7A11 expression exhibit excessive cystine uptake, leading to aberrant disulfide bond formation, disulfide-mediated collapse of the actin cytoskeleton, and ultimately, cell death [9]. Moreover, recent findings indicate that in exhausted CD8^+^ T cells, lactate dehydrogenase B (LDHB) interacts with glucose-6-phosphate dehydrogenase (G6PD), suppressing its activity and promoting NADPH depletion, ultimately inducing disulfidptosis [17]. Given the complexity of the TME, understanding the role of disulfidptosis in different cellular components of the tumor ecosystem has become an important area of investigation.

Tumor-associated macrophages (TAMs) constitute a major component of the immune infiltrate within the TME, accounting for approximately 50% of the tumor mass [18,19,20]. Substantial evidence indicates that TAMs actively contribute to tumor progression by promoting angiogenesis, remodeling the extracellular matrix, supporting cancer cell proliferation and metastasis, and mediating immunosuppression [21,22]. Clinically, high levels of TAM infiltration are consistently associated with poor prognosis and reduced survival in multiple cancer types [23,24]. A key feature underlying the accumulation and persistence of TAMs within the metabolically hostile TME is their remarkable resistance to cell death. This survival advantage is largely supported by metabolic reprogramming, which allows TAMs to adapt to conditions of nutrient deprivation and the accumulation of metabolic waste [4]. To meet their bioenergetic and biosynthetic demands under nutrient-limited circumstances, TAMs preferentially engage in aerobic glycolysis but can flexibly switch to alternative energy sources such as lactate and fatty acids when necessary [4,25]. Moreover, TAMs enhance glutathione biosynthesis to sustain redox homeostasis and counteract oxidative stress, thereby suppressing ferroptosis and further reinforcing their survival capacity [26]. In response to hypoxia, TAMs stabilize hypoxia-inducible factor 1α (HIF-1α), triggering adaptive responses, including enhanced glycolysis, promotion of angiogenesis, and secretion of immunosuppressive mediators [27,28]. These coordinated metabolic adaptations collectively enable TAMs to evade multiple forms of cell death, maintain their functional plasticity, and support tumor progression. However, whether TAMs possess common regulatory mechanisms that govern multiple forms of cell death, including disulfidptosis, remains unclear.

AMP-activated protein kinase (AMPK) is a highly conserved serine/threonine kinase that functions as a central regulator of cellular energy homeostasis [29,30]. In response to energetic stress, AMPK is activated and modulates multiple metabolic processes, including lipid metabolism, glucose metabolism, amino acid metabolism, autophagy, and mitochondrial function [31,32]. Previous studies have shown that AMPK promotes the differentiation of myeloid-derived suppressor cells into TAMs and enhances their immunosuppressive functions [33]. However, the role of AMPK in regulating TAM survival and susceptibility to cell death within the metabolically dynamic TME remains incompletely understood.

Here, through integrated multi-omics analyses and experimental validation, we show that increased AMPK activation during tumor progression is associated with transcriptomic and proteomic features indicative of reduced susceptibility of TAMs to multiple forms of cell death. Conditional deletion of AMPK in TAMs reshapes the expression of cell death-related genes and is associated with increased apoptosis, ferroptosis, and notably, disulfidptosis, a recently characterized form of regulated cell death. Clinical correlation analyses revealed that AMPK activity in TAMs was inversely associated with disulfidptosis-, ferroptosis-, and apoptosis-related gene signatures. Furthermore, tumors characterized by concurrent enrichment of AMPK signaling and TAM infiltration exhibited lower cell death-related signature scores, which were associated with a more malignant phenotype. Collectively, our findings suggest that AMPK activity is associated with TAM survival and tumor progression and with reduced susceptibility to multiple forms of cell death, including disulfidptosis. These results support a role for AMPK in the metabolic adaptation and survival of TAMs and suggest its potential as a therapeutic target for cancer intervention.

## 2. Results

### 2.1. Decreased Cell Death Signatures in TAMs Correlate with Advancing Tumor Stage

**Figure 1:**
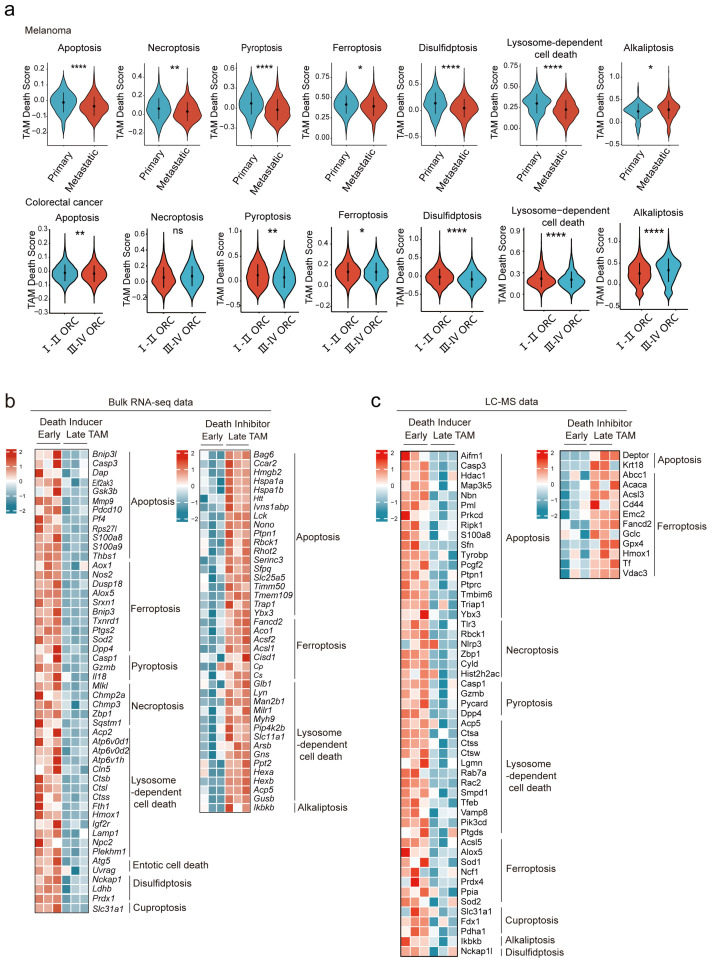
Decreased cell death signatures in TAMs correlate with advancing tumor stage. (**a**) The signature score of the death-associated gene set in human early and late-stage melanoma and colorectal cancer TAMs. *p* values were calculated by two-sided Wilcoxon rank-sum test. (**b**) Heatmap of death-inducing (death inducer) and death-inhibiting (death inhibitor) genes in early-stage (day 8) and late-stage (day 15) TAMs from B16 tumor-bearing mice. (**c**) Heatmap of death-inducing (death inducer) and death-inhibiting (death inhibitor) proteins in early-stage (day 8) and late-stage (day 15) TAMs from B16 tumor-bearing mice. Data are presented as mean ± SEM. * *p* < 0.05; ** *p* < 0.01; **** *p* < 0.0001; ns, not significant.

To investigate whether TAMs progressively exhibit molecular features associated with resistance to cell death during tumor progression, we analyzed publicly available single-cell transcriptomic datasets from clinical tumor samples [34,35]. Based on relevant published literature and the KEGG database, we curated gene sets representing various cell death modalities and subsequently performed gene set scoring for multiple cell death-related pathways in tumor-infiltrating TAMs [9,17,36,37,38,39]. This analysis revealed a stepwise decline in cell death-related signatures with advancing tumor stage. Compared with TAMs from primary melanoma, those from metastatic lesions exhibited significantly lower scores across various cell death modalities, including apoptosis, pyroptosis, necroptosis, disulfidptosis, lysosome-dependent cell death, and ferroptosis. A similar trend was observed in colorectal cancer, where TAMs from late-stage tumors displayed significantly lower cell death-related pathway scores compared to those from early-stage tumors (Figure 1a). These transcriptomic observations were further supported by functional validation in both MC38 and B16 subcutaneous tumor models, where TAMs from late-stage (day 15) tumors exhibited a significantly lower proportion of Annexin V^+^ apoptotic cells than those from early-stage (day 8) tumors (Figure S1a,b). Bulk RNA-seq analysis of TAMs revealed that late-stage TAMs showed downregulation of pro-death genes involved in apoptosis, pyroptosis, necroptosis, and disulfidptosis, along with increased expression of genes associated with negative regulation of apoptosis and ferroptosis (Figure 1b and Figure S1c). Consistently, late-stage TAMs exhibited reduced enrichment of gene sets positively regulating entotic cell death, apoptosis, pyroptosis, and disulfidptosis, whereas gene sets involved in the negative regulation of these pathways were enriched (Figure S1d). Re-analysis of our previously generated proteomic dataset corroborated these findings, revealing that late-stage TAMs had significantly reduced expression of proteins involved in multiple cell death pathways, along with increased levels of ferroptosis-inhibitory proteins (Figure 1c) [40]. These data indicate that TAMs progressively exhibit transcriptomic, proteomic, and functional features associated with reduced susceptibility to multiple forms of cell death during tumor progression.

### 2.2. Enhanced AMPK Signaling Correlates with Reduced Cell Death-Related Signatures in TAMs During Tumor Progression

**Figure 2:**
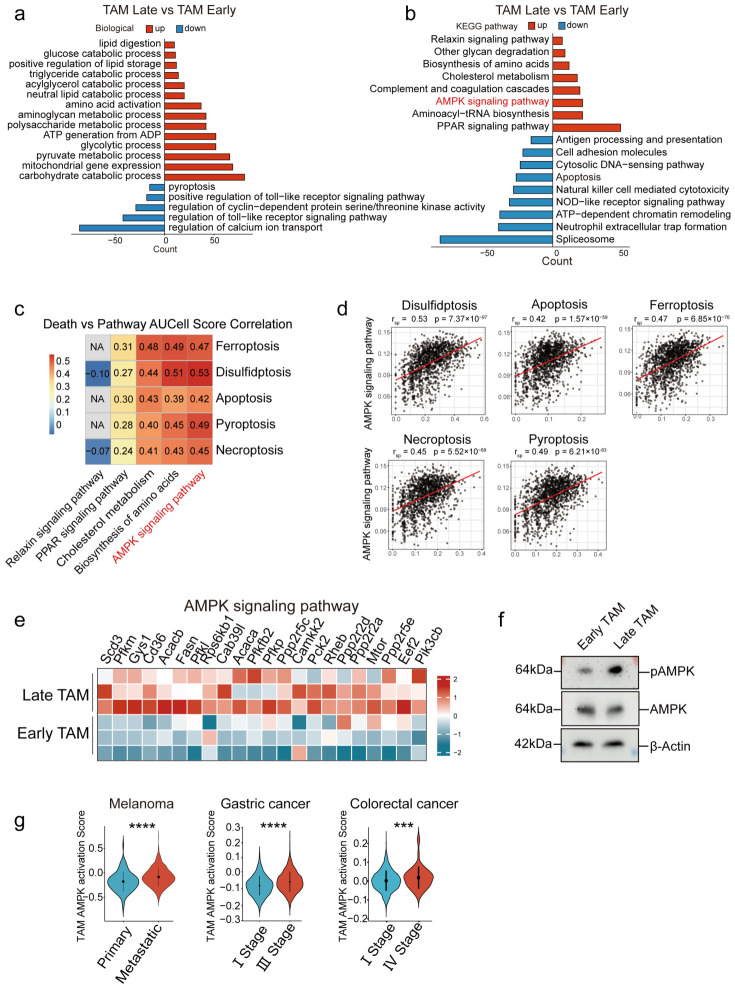
Enhanced AMPK signaling correlates with reduced cell death-related signatures in TAMs during tumor progression. (**a**) Bar plot of enriched biological process terms of DEPs in the early and late-stage TAMs derived from B16 tumor-bearing mice. (**b**) Bar plot of enriched KEGG pathways of DEPs in the early and late-stage TAMs derived from B16 tumor-bearing mice. (**c**) Heatmap of correlation scores between TAM cell death modalities and signaling pathways. (**d**) Correlation between AMPK signaling pathway and TAM cell death modalities. (**e**) Heatmap of AMPK signaling pathway activation-related proteins. (**f**) Western blot showing the expression level of total and phospho-Ampkα in TAMs sorted from B16 tumor-bearing mice in the early (day 8) and late stages (day 15). (**g**) The signature score of the AMPK signaling pathway activation gene set in human early and late-stage melanoma, colorectal cancer and gastric cancer TAMs. *p* values were calculated by two-sided Wilcoxon rank-sum test. Data are presented as mean ± SEM. *** *p* < 0.001; **** *p* < 0.0001.

To explore the molecular features associated with TAM susceptibility to cell death during tumor progression, we performed signaling pathway analyses comparing early and late-stage TAMs. GO analysis revealed extensive metabolic reprogramming in TAMs during tumor progression. Specifically, late-stage TAMs exhibited enhanced catabolic processes, including glucose, triglyceride, acylglycerol, and neutral lipid catabolism, along with increased glycolysis and pyruvate metabolism (Figure 2a). These metabolic changes may contribute to the ability of TAMs to maintain cellular homeostasis under the metabolic stress of the TME and are potentially associated with altered susceptibility to multiple forms of cell death. KEGG enrichment analysis revealed that late-stage TAMs exhibited significant downregulation of apoptosis-related pathways, while simultaneously upregulating metabolic pathways, including the AMPK signaling pathway, cholesterol metabolism, and amino acid biosynthesis (Figure 2b). To identify signaling pathways associated with altered susceptibility of TAMs to multiple forms of cell death, we performed correlation analyses based on single-cell transcriptomic data from clinical melanoma samples. The AMPK signaling pathway showed the strongest correlations with signatures of apoptosis, pyroptosis, necroptosis, ferroptosis, and disulfidptosis (Figure 2c,d). To validate the specificity of our gene set-based scoring strategy, we further assessed the potential overlap and cross-interference among apoptosis-, ferroptosis-, and disulfidptosis-related signatures. Minimal gene overlap was observed among the three gene sets. Moreover, Spearman correlation and partial correlation analyses across melanoma samples revealed only weak associations among the corresponding signature scores (Figure S2a–c). These findings indicate that the curated signatures are largely independent and support the reliability of the pathway correlation analyses.

In parallel, proteins associated with the AMPK signaling pathway were enriched in late-stage TAMs compared with early-stage TAMs (Figure 2e). This observation was further validated by Western blot analysis of TAMs isolated from B16 tumors, which demonstrated higher levels of phosphorylated AMPK (pAMPK) in late-stage compared to early-stage TAMs (Figure 2f). Consistent with these findings, tumor-infiltrating TAMs in late-stage tumors displayed significantly higher AMPK activation compared to early-stage TAMs across multiple human cancers, including melanoma, gastric cancer, and colorectal cancer (Figure 2g). Together, these findings suggest that increased AMPK activation is associated with molecular and functional features indicative of reduced susceptibility of TAMs to multiple forms of cell death during tumor progression.

### 2.3. AMPK Deficiency Increases Susceptibility to Multiple Forms of Cell Death in TAMs

**Figure 3:**
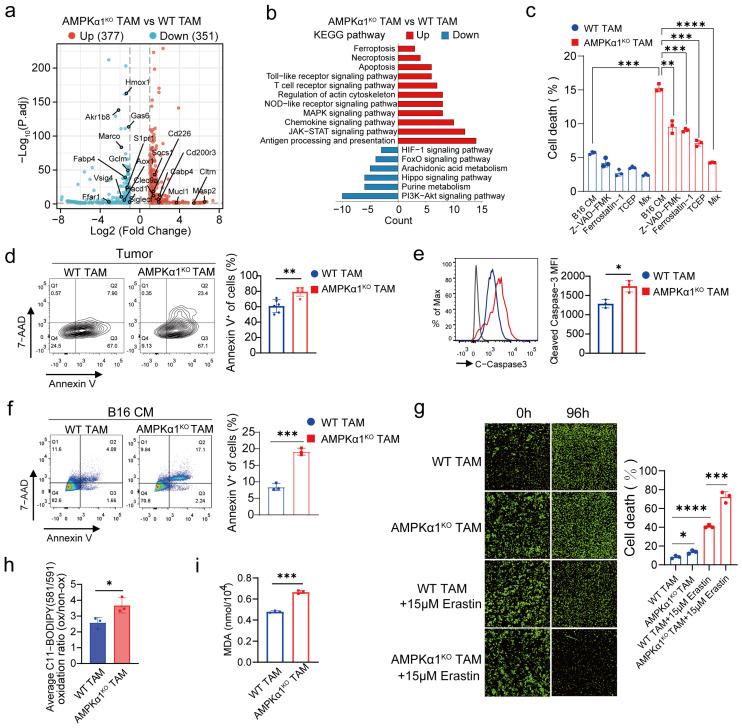
AMPK deficiency increases susceptibility to multiple forms of cell death in TAMs. (**a**) Volcano plot of DEGs in WT TAMs and AMPK^KO^ TAMs from B16 tumor-bearing mice *AMPK^fl/fl^* (*WT*) and *AMPK^fl/fl^LyZ2^cre^* (*AMPK-cKO*). (**b**) Bar plot of enriched KEGG pathway of DEGs in the TAMs from B16 tumor-bearing mice *AMPK^fl/fl^* (*WT*) and *AMPK^fl/fl^LyZ2^cre^* (*AMPK-cKO*). (**c**) Cell death in WT and AMPKα1KO TAMs induced with B16 CM in vitro and treatment with disulfide stress inhibitors TCEP (1 mM), the apoptosis inhibitor Z-VAD-FMK (10 µM), ferroptosis inhibitor Ferrostatin-1 (10 µM) and Mix inhibitor (Z-VAD-FMK (10 µM) + Ferrostatin-1 (10 µM) + (10 µM)) for 72 h. TAM death rates were assessed by PI staining and detected by High-Content Imaging System. (**d**) Flow cytometric quantification and representative plots of frequency of Annexin V+ TAMs from B16 tumor-bearing mice *AMPK^fl/fl^* (*WT*) and *AMPK^fl/fl^LyZ2^cre^* (*AMPK-cKO*), *n* = 6. (**e**) Flow cytometric quantification and representative plots of cleaved caspase-3 (C-Caspase3) expression in TAMs from B16 tumor-bearing mice *AMPK^fl/fl^* (*WT*) and *AMPK^fl/fl^LyZ2^cre^* (*AMPK-cKO*), *n* = 3. (**f**) Flow cytometric quantification and representative plots of frequency of Annexin V^+^ TAMs in *WT* and *AMPKα1^KO^* TAMs induced with B16 CM in vitro for 3 days. (**g**) Cell death quantification and representative images of GFP^+^ WT TAMs and GFP^+^ AMPKα1^KO^ TAMs induced with B16 CM and ferroptosis-inducer erastin (15 μM). (**h**) C11-BODIPY (a marker of lipid peroxidation) quantification showing lipid peroxidation accumulation in WT and AMPKα1^KO^ TAMs induced with B16 CM in vitro for 3 days. (**i**) Quantification of MDA in WT and AMPKα1^KO^ TAMs induced with B16 CM in vitro for 3 days. Data are presented as mean ± SEM. * *p* < 0.05; ** *p* < 0.01; *** *p* < 0.001, **** *p* < 0.0001.

To investigate the role of AMPK in regulating TAM susceptibility to cell death during tumor progression, we generated myeloid-specific AMPK knockout mice *AMPK^fl/fl^LyZ2^cre^* (*AMPK-cKO*) along with their littermate control mice *AMPK^fl/fl^ (WT)*, and established a B16 tumor-bearing mouse model. Bulk RNA-seq of sorted tumor-infiltrating TAMs, followed by principal component analysis, revealed a clear transcriptomic separation between AMPKα1^KO^ TAMs and WT TAMs (Figure S3a). Among 728 differentially expressed genes, KEGG enrichment analysis highlighted the upregulation of apoptosis, ferroptosis, and necroptosis pathways in AMPKα1^KO^ TAMs (Figure 3a,b). Further analysis showed that AMPK deficiency was associated with reduced expression of genes involved in the negative regulation of apoptosis, ferroptosis, and lysosome-related cell death, while genes involved in promoting these pathways were upregulated (Figure S3b–d). Notably, AMPKα1^KO^ TAMs also exhibited increased expression of necroptosis, pyroptosis, and disulfidptosis-related genes (Figure S3e–g).

To further investigate these observations, we established an in vitro TAM model by stimulating bone marrow-derived macrophages (BMDMs) with B16 conditioned medium (CM), and used this model to determine which cell death inhibitors could suppress TAM death induced by AMPK deficiency. The results showed that the ferroptosis inhibitor Ferrostatin-1, the apoptosis inhibitor Z-VAD-FMK, and the disulfidptosis inhibitor TCEP each partially rescued TAM death induced by AMPKα1 deficiency (Figure 3c). Combined treatment with multiple cell death inhibitors significantly reduced the death of AMPKα1^KO^ TAMs and exerted a stronger protective effect than any individual inhibitor alone (Figure 3c). In vivo experiments showed that AMPK deficiency markedly increased the proportion of apoptotic TAMs within tumors, accompanied by a significant upregulation of the apoptotic marker cleaved caspase 3 (Figure 3d,e). In vitro experiments using B16 CM further supported increased susceptibility of AMPKα1^KO^ TAMs to apoptosis, as evidenced by significantly higher apoptotic rates upon AMPK knockout (Figure 3f). We next investigated whether AMPK deficiency alters susceptibility of TAMs to ferroptosis. AMPKα1^KO^ TAMs induced with B16 CM displayed heightened sensitivity to erastin-induced ferroptotic death (Figure 3g), accompanied by increased lipid peroxidation, elevated malondialdehyde (MDA) levels (Figure 3h,i), and reduced expression of the ferroptosis-suppressing antioxidant enzyme glutathione peroxidase 4 (GPX4) (Figure S3h). Quantitative PCR (qPCR) analysis further confirmed that AMPK knockout downregulates ferroptosis-related gene expression (Figure S3i). Collectively, these findings suggest that AMPK deficiency is associated with increased susceptibility of TAMs to multiple forms of cell death during tumor progression.

### 2.4. AMPK Deficiency Is Associated with Disulfidptosis-Related Cellular Features in TAMs and Reduced Tumor Growth

**Figure 4:**
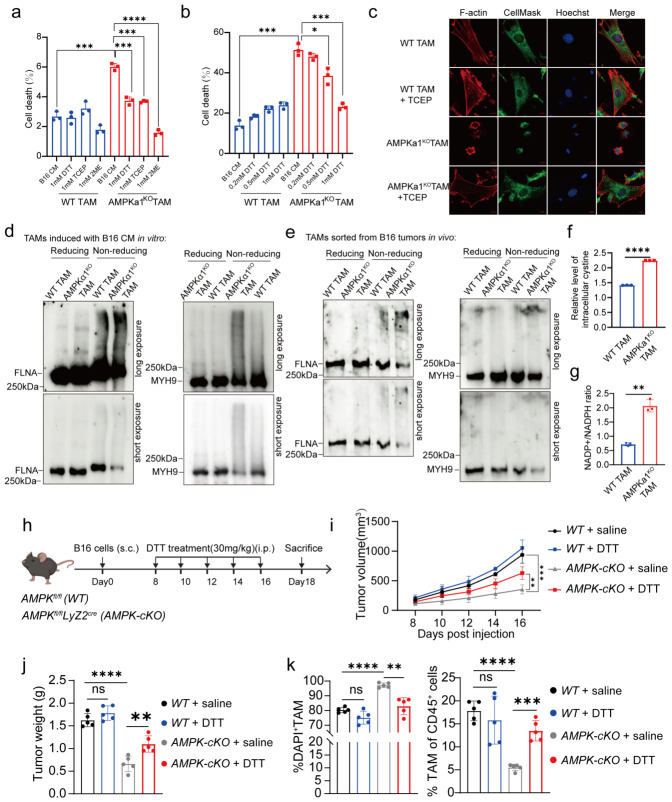
AMPK deficiency is associated with disulfidptosis-related cellular features in TAMs and reduced tumor growth. (**a**) Cell death in WT and AMPKα1^KO^ TAMs induced with B16 CM in vitro and treatment with disulfide stress inhibitors TCEP (1 mM), DTT (1 mM), 2ME (1 mM) for 72 h. TAM death rates were assessed by PI staining and detected by Flow cytometry. (**b**) Cell death in WT and AMPKα1^KO^ TAMs induced with B16 CM in vitro and treatment with disulfide stress inhibitors DTT in different concentrations for 5 days. TAM death rates were assessed by PI staining and detected by Flow cytometry. (**c**) Fluorescent staining of F-actin with phalloidin and the cell membrane with CPM^TM^ Green in WT and AMPKα1^KO^ TAM induced with B16 CM in vitro and treatment with disulfide stress inhibitors TCEP (1 mM) for 72 h. Scale bar = 20 μm. F-actin was stained with phalloidin (red), the cell membrane with CPM™ Green (green), and the nucleus with Hoechst (blue). (**d**) Non-reducing and reducing Western blot analysis of the indicated actin cytoskeleton proteins in WT and AMPKα1^KO^ TAM induced with B16 CM in vitro for 72 h. (**e**) Non-reducing and reducing Western blot analysis of the WT and AMPKα1^KO^ TAMs sorted from B16 tumor-bearing mice *AMPK^fl/fl^* (*WT*) and *AMPK^fl/fl^LyZ2^cre^* (*AMPK-cKO*). (**f**) A cystine assay kit was used to measure cystine levels in WT and AMPK^KO^ TAM induced with B16 CM in vitro for 72 h. (**g**) A kit was used to measure the ratio of NADP^+^/NADPH in WT and AMPKα1^KO^ TAMs induced with B16 CM in vitro for 3 days. (**h**) Treatment scheme for B16 tumor-bearing mouse models of *AMPK^fl/fl^* (*WT*) and *AMPK^fl/fl^LyZ2^cre^* (*AMPK-cKO*) mice. (**i**) Tumor volume was measured every two days from the eighth day after tumor cells injection in *AMPK^fl/fl^* (*WT*) and *AMPK^fl/fl^LyZ2^cre^* (*AMPK-cKO*) mice. (*n* = 5/group). (**j**) Tumor weight was measured on the day of sacrifice. (**k**) Flow cytometric quantification of frequency of DAPI^+^ TAMs and total TAMs proportion in *AMPK^fl/fl^* (*WT*) and *AMPK^fl/fl^LyZ2^cre^* (*AMPK-cKO*) mice treated with DTT or not. Data are presented as mean ± SEM. * *p* < 0.05; ** *p* < 0.01; *** *p* < 0.001; **** *p* < 0.0001; ns, not significant.

Given that disulfidptosis exhibited the strongest correlation with AMPK signaling in our prior analyses (Figure 2c,d), we next examined whether AMPK deficiency is associated with disulfidptosis-related cellular features in TAMs. To this end, we treated TAMs in an in vitro induction system with various disulfidptosis inhibitors and found that cell death in AMPKα1^KO^ TAM was partially rescued in a dose-dependent manner by multiple inhibitors, including TCEP, dithiothreitol (DTT), and 2-mercaptoethanol (2-ME) (Figure 4a,b and Figure S4a). Previous studies have shown that disulfidptosis is triggered by disulfide stress in SLC7A11^high^ tumor cells under glucose deprivation, and is characterized by cell shrinkage and F-actin cytoskeletal contraction [9]. Consistent with this, phalloidin staining revealed that AMPKα1^KO^ TAMs exhibited marked cell shrinkage and F-actin collapse compared to WT TAMs, an effect that was significantly reversed by TCEP treatment (Figure 4c). It has also been reported that tumor cells undergoing disulfidptosis exhibit aberrant disulfide bonding within actin cytoskeleton proteins, which alters their electrophoretic mobility under non-reducing conditions. This is typically characterized by reduced migration, a pronounced smearing pattern, and, in some cases, the formation of high-molecular weight aggregates near the stacking gel [9]. Based on these observations, we performed non-reducing Western blot analysis to assess disulfide bond-associated alterations in cytoskeletal proteins. In an in vitro TAM induction system, non-reducing immunoblot analysis revealed that actin cytoskeleton-associated proteins, including FLNA and MYH9, exhibited markedly reduced electrophoretic mobility accompanied by a characteristic smearing pattern in AMPKα1^KO^ TAMs, along with a notable decrease in basal protein levels. In contrast, these aberrant migration patterns were completely abolished under reducing conditions in AMPKα1^KO^ TAMs, indicating the formation of extensive intermolecular disulfide bonds within cytoskeletal proteins (Figure 4d)**.** To further validate these findings in vivo, we established *AMPK^fl/fl^LyZ2^cre^* (*AMPK-cKO*) and *AMPK^fl/fl^* (*WT*) B16 tumor-bearing mouse models. Primary tumor-infiltrating TAMs were isolated and subjected to non-reducing immunoblotting to assess disulfide bond formation. Consistently, AMPKα1 deficiency in TAMs led to the accumulation of FLNA and MYH9 as high-molecular weight aggregates retained near the stacking gel, accompanied by a pronounced reduction in their monomeric forms (Figure 4e). In addition, we found that AMPK deficiency led to a marked accumulation of cystine, a key driver of disulfidptosis, in TAMs. (Figure 4f). Consistent with this finding, AMPKα1^KO^ TAMs exhibited a significantly elevated NADP+/NADPH ratio, suggesting reduced NADPH availability and a diminished capacity for cystine reduction, which may contribute to intracellular cystine accumulation (Figure 4g). Collectively, these results indicate that AMPK deficiency is associated with cystine accumulation and aberrant intermolecular disulfide bonding in cytoskeletal proteins in TAMs, features that are consistent with increased susceptibility to disulfidptosis.

To further assess the impact of AMPK deficiency-associated TAM death on tumor progression, we established multiple tumor models in *AMPK^fl/fl^* (WT) and *AMPK^fl/fl^Lyz2^cre^* (*AMPK-cKO*) mice. Tumor growth was significantly slower in *AMPK-cKO* mice (Figure S5a–c), accompanied by a marked reduction in TAM abundance (Figure S4d), suggesting that AMPK is critical for TAM survival in the tumor microenvironment. To further investigate whether AMPK knockout-induced disulfidptosis in TAMs contributes to tumor growth suppression, we established B16 tumor models treated with either saline or the disulfidptosis inhibitor DTT (Figure 4h). Notably, DTT treatment substantially diminished the tumor growth difference between *AMPK-cKO* mice and controls, suggesting that disulfidptosis contributes to the tumor-suppressive effects associated with AMPK deficiency (Figure 4i,g). We next assessed TAM death by DAPI staining to label dead cells. Compared with controls, *AMPK-cKO* mice exhibited a significantly higher proportion of dying TAMs, which was effectively reduced by DTT treatment (Figure 4k, left). Additionally, the decreased TAM infiltration observed in *AMPK-cKO* tumors was partially rescued by DTT administration (Figure 4k, right). Collectively, these results suggest that AMPK deficiency is associated with cystine accumulation and aberrant disulfide bonding in cytoskeletal proteins in TAMs, which is likely to contribute to increased susceptibility to disulfidptosis-related cellular damage.

### 2.5. High AMPK Activity in TAMs Accompanies Reduced Cell Death-Related Signatures and Poor Prognosis in Cancer Patients

**Figure 5:**
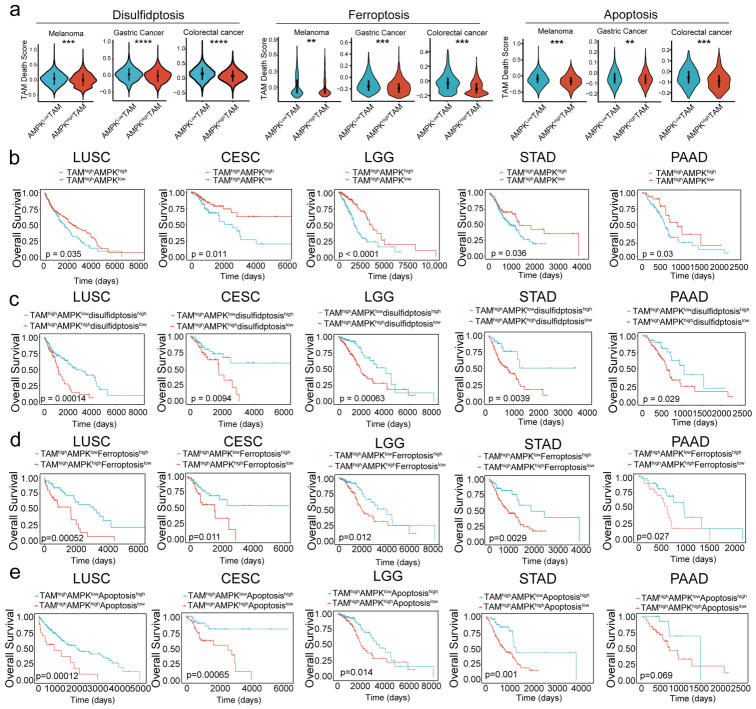
High AMPK activity in TAMs accompanies reduced cell death-related signatures and poor prognosis in cancer patients. (**a**) Signature scores of the disulfidptosis, ferroptosis and apoptosis-associated gene set in TAMs with high and low AMPK activation across human melanoma, colorectal, and gastric cancers. *p* values were calculated by two-sided Wilcoxon rank-sum test. (**b**–**e**) Kaplan–Meier survival analysis in a pan-cancer TCGA cohort stratified by TAMs infiltration, AMPK activation status, and disulfidptosis (ferroptosis or apoptosis) levels. Data are presented as mean ± SEM. ** *p* < 0.01; *** *p* < 0.001; **** *p* < 0.0001.

To investigate whether AMPK activity is associated with regulation of cell death programs in TAMs, we analyzed single-cell RNA-sequencing datasets from melanoma, colorectal cancer, and gastric cancer. Across all three tumor types, TAMs with high AMPK activity consistently displayed significantly reduced expression of disulfidptosis-, ferroptosis-, and apoptosis-related gene signatures compared with their AMPK-low counterparts (Figure 5a). These results suggest that high AMPK activity in TAMs is associated with reduced disulfidptosis, ferroptosis, and apoptosis in clinical tumor samples. To further assess the clinical relevance of the association between AMPK activity and cell death programs in TAMs, we performed stratified analyses using TCGA datasets. Kaplan–Meier survival analyses demonstrated that tumors characterized by both high AMPK signaling and abundant TAM infiltration exhibited lower expression of disulfidptosis-, ferroptosis-, and apoptosis-related gene signatures. Notably, this phenotype was associated with poorer overall survival and increased tumor malignancy (Figure 5b–e). Collectively, these findings suggest that AMPK activation in TAMs is associated with reduced expression of gene signatures related to multiple forms of cell death, including disulfidptosis, ferroptosis, and apoptosis, across diverse human cancers. Clinically, tumors characterized by high AMPK signaling and abundant TAM infiltration exhibit reduced cell death signatures and are associated with poorer patient survival and increased malignancy. These results suggest that high AMPK activity in TAMs is associated with reduced cell death-related signatures and with clinical features linked to tumor progression and adverse outcomes.

## 3. Discussion

TAMs are a major immune cell population in the TME, accounting for about 50% of the tumor mass [18,19,20]. To adapt to the nutrient-deprived and metabolically stressed TME, TAMs resist cell death by utilizing various metabolic reprogramming pathways [2,41]. However, the key molecular regulators underlying TAM survival during tumor progression remain incompletely understood. Here, we found that TAMs progressively acquired transcriptomic, proteomic, and functional features associated with reduced susceptibility to multiple forms of cell death during tumor progression. Integrated multi-omics analyses further showed that increased AMPK activity was associated with this phenotype. Conditional deletion of AMPK in TAMs altered the expression of cell death-related genes and was associated with increased apoptosis, ferroptosis, and notably, disulfidptosis. Clinically, AMPK activity in TAMs inversely correlates with the expression of disulfidptosis-, ferroptosis-, and apoptosis-related gene signatures. Moreover, tumors characterized by both high AMPK signaling and abundant TAM infiltration exhibited lower disulfidptosis, ferroptosis, and apoptosis scores, which were associated with increased tumor malignancy and poorer clinical outcomes. Collectively, these findings suggest that AMPK activity is associated with TAM survival and reduced susceptibility to multiple forms of cell death, including disulfidptosis, and may represent a potential target for metabolic modulation of TAMs in cancer.

The TME constitutes a metabolically dynamic ecosystem characterized by hypoxia, nutrient deprivation (e.g., glucose, amino acids, glutamine), and the accumulation of cytotoxic metabolites, including lactate, adenosine, fatty acids, cholesterol, and reactive oxygen species [1,2,42]. These conditions drive intense intercellular competition for limited resources, forcing cellular metabolic reprogramming to sustain survival and functionality [4,41]. Maladaptive cells become susceptible to regulated metabolic death modalities—such as disulfidptosis, ferroptosis, cuproptosis, lysosome-dependent cell death, alkaliptosis, and ammonia-induced cell death—mediated by nutrient scarcity, metabolite toxicity, or disrupted metal homeostasis [6,8,9,10,12,13,39,43]. Notably, nutrient deprivation and lactate accumulation drive activated CD8^+^ T cells toward exhaustion within the TME [44,45]. CD36-mediated excessive fatty acid uptake induces lipid peroxidation and ferroptosis in CD8^+^ T cells [39]. Accelerated glutaminolysis generates cytotoxic ammonia accumulation, which triggers ammonia-induced cell death in CD8^+^ T cells [12]. Natural killer (NK) cells that internalize high concentrations of lactate experience decreased intracellular pH, reduced ATP levels, and impaired energy metabolism, ultimately suppressing cytotoxic activity while promoting apoptosis [43]. In this intricate and dynamic TME, TAMs differentiate into distinct metabolic phenotypes, exhibiting remarkable metabolic adaptability and actively supporting tumor cells. For instance, in lung adenocarcinoma, TAMs are regulated by GM-CSF secreted by cancer cells within the TME, which sustains their survival while simultaneously prompting TAMs to release free fatty acids and cholesterol to support tumor cell metabolism [46]. In glioblastoma, TAMs recycle cholesterol-rich myelin debris into lipids to sustain their own survival, while also transferring these lipids to glioblastoma cells as fuel [47]. Consistently, our study demonstrates that during tumor progression, TAMs progressively exhibit transcriptomic, proteomic, and functional features associated with reduced susceptibility to multiple forms of cell death, including apoptosis, ferroptosis, and disulfidptosis. It should be noted that our conclusion that TAMs progressively acquire transcriptomic and proteomic features associated with reduced susceptibility to multiple forms of programmed cell death during tumor progression, accompanied by increased AMPK activity, is drawn mainly from bioinformatic analyses of transcriptomic and proteomic data. Collectively, these findings suggest that the pronounced metabolic adaptability of TAMs may contribute to their reduced susceptibility to diverse forms of cell death, thereby supporting their persistence and tumor-promoting functions within the metabolically hostile TME.

AMPK functions as a cellular sensor of energy status and a central regulator of metabolic homeostasis, thereby contributing to the maintenance of cellular energy balance [31]. Although activation of AMPK under energetic stress has been reported to protect tumor cells from ferroptosis, its role in regulating cell death in TAMs remains poorly understood [48]. Our findings suggest that AMPK activity is associated with the regulation of multiple cell death pathways in TAMs. First, AMPK activity increased markedly in TAMs during tumor progression, consistent with its potential responsiveness to metabolic changes within the TME. Second, integrated multi-omics analyses and experimental validation in mouse models showed that AMPK deficiency increased the susceptibility of TAMs to multiple forms of cell death, including apoptosis, ferroptosis, and disulfidptosis. Notably, AMPK deficiency was associated with increased susceptibility to disulfidptosis-related cellular features in TAMs and was accompanied by impaired tumor progression. Consistent with these experimental findings, clinical analyses showed that lower AMPK activity in TAMs, together with a stronger disulfidptosis-associated signature, was correlated with prolonged overall survival. Taken together, these experimental and clinical findings support a role for AMPK in association with reduced susceptibility of TAMs to multiple forms of cell death during tumor progression, with disulfidptosis emerging as a particularly relevant form of cell death linked to tumor control and patient outcomes.

Among the multiple forms of cell death associated with AMPK activity in TAMs, disulfidptosis warrants particular attention due to its association with tumor control and patient outcomes. As a recently identified form of regulated cell death, disulfidptosis has also been implicated in T-cell biology, with recent studies showing that LDHB promotes this process in T cells [17]. Our study extends these observations to TAMs and suggests a potential basis for targeting TAMs through metabolic intervention. However, the molecular basis underlying the relationship between AMPK activity and disulfidptosis in TAMs remains incompletely understood. We observed that AMPK deficiency in TAMs was accompanied by reduced NADPH production and increased accumulation of cystine and related metabolites. Given that NADPH is largely generated through the pentose phosphate pathway, in which glucose-6-phosphate dehydrogenase (G6PD) serves as the rate-limiting enzyme [49], G6PD may represent a potential link between AMPK activity, redox homeostasis, and disulfidptosis. This possibility is also consistent with recent evidence showing that G6PD suppresses disulfidptosis in CD8^+^ T cells [17]. Nevertheless, the regulation of G6PD by AMPK appears to be highly context dependent. Depending on the cell type and metabolic state, AMPK has been reported to enhance G6PD expression or activity [50], exert limited effects [51], or indirectly suppress its function [52]. Thus, the AMPK–G6PD relationship may not follow a uniform regulatory pattern across biological contexts. Whether AMPK modulates G6PD expression or activity in TAMs, and whether this contributes to altered NADPH availability, redox imbalance, and disulfidptosis, requires further investigation.

In summary, through integrated single-cell and bulk transcriptomic, proteomic, and experimental analyses, this study identifies AMPK as an important factor associated with TAM survival during tumor progression. We found that TAMs progressively acquired transcriptomic and proteomic features associated with reduced susceptibility to multiple forms of cell death, accompanied by increased AMPK activity. Genetic ablation of AMPK altered cell death-related programs in TAMs and increased their susceptibility to apoptosis, ferroptosis, and disulfidptosis. Among these processes, disulfidptosis emerged as a particularly relevant event associated with TAM depletion following AMPK deficiency. Functional in vivo studies further suggested that enhanced susceptibility to disulfidptosis is associated with reduced TAM abundance and impaired tumor growth in AMPK-deficient mice. Consistent with these experimental findings, clinical analyses showed that elevated AMPK activity in TAMs was associated with lower disulfidptosis-, ferroptosis-, and apoptosis-related signatures, as well as poorer patient outcomes. Collectively, these findings support a model in which AMPK activity is associated with TAM persistence and tumor progression and with reduced susceptibility to multiple forms of cell death, including disulfidptosis. Although our data support a functional association between AMPK activity, TAM survival, and disulfidptosis, the molecular basis underlying this relationship remains incompletely understood. Further investigation of this regulatory relationship and its connection with established disulfidptosis-associated pathways may provide new opportunities for therapeutically targeting TAMs in cancer.

## 4. Materials and Methods

### 4.1. Mice and Tumor Models

*AMPK^fl/fl^* (*WT*) mice were obtained from the Innovation Institute for Integration of Medicine and Engineering, WCH, SCU. Female C57BL/6 mice (5 weeks) were purchased from Beijing Vital River Laboratory (Beijing, China). *AMPK^fl/fl^* (*WT*) mice were crossed with LysM^Cre^ mice to generate macrophage-specific *AMPK^fl/fl^Lyz2^cre^* (*AMPK-cKO*) mice. Mice were genotyped from tail biopsy using the primers listed in the Supplementary Table S1. List of primer. All mice were on a C57BL/6 background and were maintained at the SPF facilities of the Beijing Institute of Lifeomics. Mice aged 5 weeks were used for animal experiments in this study.

B16-F10 (5 × 10^5^) cells, MC38 cells (5 × 10^5^), and Hep1-6 (1 × 10^6^) cells were implanted subcutaneously in *AMPK^fl/fl^* (*WT*), *AMPK^fl/fl^Lyz2^cre^* (*AMPK-cKO*) and C57BL/6J female mice. The tumor growth was measured using calipers every 2 days. Tumor volume was calculated as follows: V = (0.5 × length × width × width). Mice were administered the drug DTT via intraperitoneal injection once every two days. The dosage of DTT injection in mice was 30 mg/kg. Tumors were harvested and isolated into single cells from euthanized mice at the indicated time points, and immune cells were analyzed by flow cytometry.

### 4.2. Cell Lines and Cell Culture

B16-F10 (ATCC^®^ CRL-6475™) and Hepa1-6 (ATCC^®^ CRL-1830™) were obtained from the American Type Culture Collection (ATCC, Manassas, VA, USA). The MC38 cell line was purchased from Kerafast (Catalog No. ENH204-FP, Boston, MA, USA). All cells were cultured in DMEM (Cat#: C11330500BT, Gibco, Carlsbad, CA, USA) supplemented with 10% fetal bovine serum (Cat#: A5669701, Gibco, Carlsbad, CA, USA) and 1% penicillin–streptomycin (Gibco) at 37 °C in a 5% CO_2_ atmosphere. All cell lines were authenticated by the suppliers using short tandem repeat (STR) profiling, routinely tested negative for mycoplasma, and used within 10 passages after thawing.

### 4.3. Bone Marrow-Derived Macrophages (BMDMs) Culture

Femora and tibiae of C57BL/6, *AMPK^fl/fl^* (*WT*)*, AMPK^fl/fl^Lyz2^cre^* (*AMPK-cKO*) mice were harvested and the bone marrow cells from all bones were flushed out. Then after centrifuging cells for 5 min at 500× *g*, erythrocytes were eliminated using red blood cell lysing buffer (Cat#: R1010, Solarbio, Beijing, China). The remaining cells were seeded in plates and incubated in complete DMEM medium with 10 ng/mL of recombinant mouse M-CSF (Cat#: 70-0161-U500,Tonbo Biosciences, San Diego, CA, USA), for 3 days.

### 4.4. Tumor Cell Suspension Preparations

B16 tumor-bearing mice were euthanized by cervical dislocation, and the tumors were obtained. The tumors were chopped and digested for 30 min at 37 °C in RPMI medium containing 10% fetal bovine serum supplemented with 2 mg/mL type IV collagenase and 0.05 mg/mL DNase I (Deoxyribonuclease I, Sigma-Aldrich, St. Louis, MO, USA), followed by filtration through a cell strainer (70 μm) to obtain single-cell suspensions. After lysing the red blood cells using RBC lysis buffer (Cat#: 555899, BD Biosciences, San Jose, CA, USA), the obtained cells were washed twice with RPMI medium and collected by centrifugation (3 min; 500 *g*; 4 °C).

### 4.5. Flow Cytometry and FACS Sorting

For flow cytometric analysis, single-cell suspension was first incubated with Fcγ receptor blocker (CD16/32, eBioscience, San Diego, CA, USA), then stained with specific mAbs. For surface marker analysis, cell pellets were stained with appropriate antibodies at 4 °C for 20–30 min. For intracellular cytokine analysis, cells were stained with the Cytofix/Cytoperm kit according to the manufacturer’s instructions (Cat# 554714, eBioscience, San Diego, CA, USA).

TAMs were identified as CD45^+^CD11b^+^Ly6C^−^Ly6G^−^F4/80^+^. The stained cells were analyzed on an LSRFortessa cell analyzer (BD Biosciences, San Jose, CA, USA). The acquired data were analyzed with FlowJo software (v10.8).

For cell purification, tumor leukocytes were isolated as described above, followed by staining for cell-surface markers. Then, TAMs were sorted by FACSAria III (BD Biosciences, San Jose, CA, USA).

### 4.6. Conditioned Medium (CM) Preparation

B16 cells were cultured in a 37 °C constant temperature incubator containing 5% CO_2_ and grown to 80% confluence. Then, the cells were washed with PBS twice and incubated in serum-free medium for 24 h. Subsequently, CM was collected and filtered through a 0.22 μm membrane sterile filter and stored at −80 °C for subsequent experiments.

### 4.7. Cell Death Assays

To measure the death of TAMs, BMDMs were induced with DMEM medium containing M-CSF (10 ng/mL) in 24-well plates three days before treatment. Then, BMDMs were treated with B16 CM for 3 days and then stained with propidium iodide (PI; Cat#: P3566, Life Technologies, Carlsbad, CA, USA) according to the manufacturer’s protocol. The cell viability was then assessed by flow cytometry analysis through LSRFortessa cell analyzer (BD Biosciences). In an alternative method, BMDMs were treated with B16 CM for 3 days, followed by staining with 2.5 µg/mL of propidium iodide (Cat#: 2549278, Invitrogen, Carlsbad, CA, USA). After 108 h, the plate was scanned for fluorescence and phase-contrast images (four image fields per well) using a fluorescence microscope (Nikon TiE, Tokyo, Japan). Image analysis was performed using the software provided with the microscope, and dead cell quantification was carried out using ImageJ software (v1.53a).

### 4.8. Quantitative RT-PCR

Total RNA was extracted from treated BMDMs and TAMs with TRIzol reagent by a Direct-zol RNA MiniPrep Kit (Cat#: R2050, Zymo Research, Irvine, CA, USA), following with the reverse transcription of 1 μg of total RNA to cDNA by the HiScript III All-in-one RT SuperMix Perfect for qPCR ((Cat#: R333-01, Vazyme, Nanjing, China). Next, qRT-PCR was performed using a SYBR Green Realtime PCR Master Mix (Cat#: QPK-201, TOYOBO, Shanghai, China). Relative mRNA expression (fold change) was calculated using the 2^−ΔΔCT^ methodology and normalized to β-actin levels. The sequences of the primers used in this study are provided in the Supporting Information. Each experiment was conducted independently at least three times.

### 4.9. Western Blot (WB) Analysis

RIPA lysis buffer containing 1% protease and phosphatase inhibitors was used to extract proteins from cells. Protein quantification was carried out using a BCA protein assay kit. Then, 10–12% sodium dodecyl sulfatepolyacrylamide gel electrophoresis (SDS-PAGE) was performed to resolve the protein samples, and a polyvinylidene fluoride (PVDF) membrane was used for protein electrotransfer, followed by membrane blocking in 5% milk for 45 min. After that, the membrane was incubated with the corresponding primary antibodies at 4 °C overnight and incubated with HRP conjugated secondary antibodies at room temperature for another 1 h. The protein bands in the membrane were examined and visualized on an LAS 500 images Imaging System.

### 4.10. Non-Reducing Western Blotting

Protein samples were combined with NuPAGE LDS Sample Buffer (4×) (Cat#: NP0007, Life Technologies, Carlsbad, CA, USA) without any reducing agents and split into two aliquots for each sample. While one aliquot was for non-reducing analysis, beta-mercaptoethanol at 2% final concentration was added to the other aliquot for reducing analysis. All samples were incubated at 70 °C for 10 min before SDS-PAGE analysis. The primary antibodies and concentrations used for Western blotting were as follows: Anti-xCT antibody (1;1000, Cat#ab307601, Abcam Technology, MA, USA), Myosin IIa (MYH9) (1:500, Cat#3403S, Cell Signaling Technology, Danvers, MA, USA), Talin-1 (TLN1) (1:500, Cat# 4021S, Cell Signaling Technology, Danvers, MA, USA), GPX4 (1:1000, Cat#ab125066, Abcam Technology, MA, USA), Phospho-AMPKα (Thr172) (1:1000, Cat#2535, Cell Signaling Technology, Danvers, MA, USA), AMPKα (1:1000, Cat#2532, Cell Signaling Technology, Danvers, MA, USA), GLUT1 (1:1000; Cat#12939, Cell Signaling Technology, Danvers, MA, USA), G6PD (1:1000, Cat#12263, Cell Signaling Technology, Danvers, MA, USA).

### 4.11. Measurement of Lipid Peroxidation

Measurements of lipid peroxidation using BODIPY 581/591 C11 (Cat#: S0043S, Beyotime Biotechnology, Shanghai, China). Briefly, cells in 12-well plates were stained with BODIPY 581/591 C11 dye (final concentration of 1 µM) for 30 min following treatments. The cells were then collected and washed once with PBS, followed by FACS analysis in cold PBS. Fluorescence in FITC and PE channel in live cells was captured and plotted using the FlowJo software (v10.8).

### 4.12. Measurement of Cystine

Cystine concentration was detected via ELISA kit (double-antibody sandwich method) (Cat#: YS16037B, YaJi Biological, Shanghai, China). WT TAMs and AMPK^KO^ TAMs were pretreated and diluted, then incubated with capture antibody-coated plates at 37 °C for 30 min. After washing, HRP-labeled secondary antibody was added for another 37 °C incubation (30 min). TMB was used for color development (15 min, 37 °C, dark), and reaction was terminated with dilute sulfuric acid. OD value was measured at 450 nm, and concentration was calculated via standard curve (0.39–25 µMoL/L) with fitting software.

### 4.13. Measurement of MDA

The pre-treated BMDMs were collected and homogenized in ice-cold Extraction Buffer (Cat#: KTB1050, Abbkine, Wuhan, China), and then centrifuged at 10,000 *g* for 10 min at 4 °C to obtain the supernatants. To assess the levels of MDA in WT TAMs and AMPK-KO TAMs, the MDA Assay Kit (Cat#: KTB1050, Abbkine, Wuhan, China) was performed according to the manufacturer’s protocol, and then the reactive substances were measured at 532 nm and 600 nm with a MultiSkan Sky microplate reader (Thermo Fisher Scientific, Waltham, MA, USA). The MDA content was calculated based on the absorbance values measured at 532 nm and 600 nm, normalized to the cell number.

### 4.14. F-Actin Staining

BMDMs were cultured on round coverslips in 6-well plates and induced with M-CSF for 3 days, followed by treatment with B16 CM for specified durations. Cells were then fixed with 4% paraformaldehyde at 4 °C for 20 min and permeabilized in PBS buffer containing 0.3% sodium deoxycholate and 0.3% Triton X-100 for 30 min at room temperature. After blocking with 5% BSA in PBS at 4 °C for 1 h, samples were incubated with phalloidin (Cat#: KTC4009, Abbkine, Wuhan, China; diluted 1:200 in incubation buffer composed of 2.5% BSA and 0.05% Triton X-100 in DPBS) for 1 h at room temperature. Nuclei were counterstained with Hoechst (1:1000 dilution, Cat#: 62249, Invitrogen, Carlsbad, CA, USA) at room temperature, and coverslips were mounted using ProLong Gold Antifade Mountant (Cat#: P10144, Invitrogen, Carlsbad, CA, USA). Images were acquired using a laser-scanning confocal microscope (LSM880 with Airyscan/ELYRA S.1 system, Carl Zeiss Microscopy GmbH, Jena, Germany).

### 4.15. Immunofluorescence Staining

Cells were cultured on round coverslips in 6-well plates, fixed with 4% paraformaldehyde for 30 min, and then incubated with 0.3% Triton X-100 for 15 min to enhance cell membrane permeability. Subsequently, the cells were incubated with 5% bovine serum albumin for 40–60 min. After washing with PBS three times, the cells were incubated with the corresponding primary antibodies overnight at 4 °C, followed by incubation with the fluorescently labeled secondary antibody for 1 h, and 15 min of incubation with Hoechst at room temperature in the dark. Coverslips were mounted using ProLong Gold Antifade Mountant (Cat#: P10144, Invitrogen, Carlsbad, CA, USA) and Laser-Scanning Confocal Microscope (LSM880 with Airyscan/ELYRA S.1 system, Carl Zeiss Microscopy GmbH, Jena, Germany) was used to observe the fluorescence staining.

### 4.16. Bulk RNA Sequencing

The 5 × 10^5^ TAMs of each sample were sorted from the tumors of *AMPK^fl/fl^*, *AMPK^fl/fl^Lyz2^cre^* mice after subcutaneous implantation of B16-F10 cells 15 day. Bulk RNA-Seq was performed by Novogene. RNA samples were sequenced using Illumina NovaSeq 6000 (Illumina, San Diego, CA, USA) according to the standard protocol. Raw data were filtered to obtain clean data. Analysis was conducted using the clean data. Reads were mapped to the mouse genome GRCh38 with HISAT2 and were quantified with the featureCounts function.

### 4.17. Bulk RNA-Seq Data Analysis

The DEGs between WT TAM and AMPK-KO TAM were calculated by the DESeq2 package, then screened with *p*-value < 0.05 and |log2FC| > 1 to identify the differences. Furthermore, Gene Ontology (GO) function and Kyoto Encyclopedia of Genes and Genomes (KEGG) functional enrichment analysis was performed using the Clusterprofiler (V3.16.1) package. Gene set activity for distinct cell death pathways (e.g., apoptosis, ferroptosis, necroptosis, and cuproptosis) was quantified using the IOBR package (v0.1.0).

### 4.18. Proteome Data Analysis

The raw proteomic data for early- and late-stage TAMs were obtained from the iProX repository (https://www.iprox.cn/page/project.html?id=IPX0007435000, accessed on 25 May 2026), accessed via the dataset identifier ProteomeXchange: PXD046492. Protein intensity normalization and identification of differentially expressed proteins (DEPs) were performed using the R packages ProstaR (v1.28.0) and DAPAR (v1.26.0). Proteins exhibiting a *p*-value < 0.05 and a fold change of <−1.5 or >1.5 were classified as significant DEPs. Functional classification of these DEPs, including Gene Ontology (GO) annotation and Kyoto Encyclopedia of Genes and Genomes (KEGG) signaling pathway enrichment analysis, was conducted using the R package clusterProfiler (v4.10.0). Statistical significance was assessed using paired or unpaired *t*-tests, with *p* < 0.05 considered statistically significant.

### 4.19. Single-Cell Data Analysis

Single-cell RNA-sequencing (scRNA-seq) data from melanoma (GSE225063) colorectal cancer (GSE236581), and gastric cancer (GSE183904) were obtained from public databases. Macrophages were extracted, re-integrated, and re-clustered from the data. Data normalization was performed using the LogNormalize method in Seurat (v3.1.0) with default parameters. The top 2000 highly variable genes were identified using the vst method within the FindVariableFeatures function. Cell clustering was conducted using the FindClusters function, with the resolution parameter set to 0.8. Cluster annotations were determined based on canonical marker genes. Gene module scores were calculated using the AUCell, and statistical differences in marker gene expression and module scores were evaluated using the Wilcoxon test. Spearman’s correlation analysis was employed to assess the associations among signaling pathways.

### 4.20. Survival Analysis

Survival analysis was conducted on the TCGA-SKCM, TCGA-CESC, TCGA-LGG, TCGA-STAD, TCGA-LUSC dataset using the Kaplan–Meier method. The survival (v3.5-7) and survminer R (v0.4.9) packages were used to generate survival curves. For gene expression stratification, quantile-based thresholds were applied to categorize ssGSEA gene set enrichment scores for the TAM signature, AMPK activation, and disulfidptosis as ‘High’ or ‘Low’.

## 5. Conclusions

1. Decreased cell death signatures in TAMs correlate with advancing tumor stage.
2. Enhanced AMPK signaling correlates with reduced cell death-related signatures in TAMs during tumor progression.
3. AMPK deficiency increases susceptibility to multiple forms of cell death in TAMs
4. AMPK deficiency is associated with disulfidptosis-related cellular features in TAMs and reduced tumor growth.
5. High AMPK activity in TAMs accompanies reduced cell death-related signatures and poor prognosis in cancer patients.

## Data Availability

The data supporting the findings of this study are publicly available in the following repositories. The raw RNA-seq data generated in this study are available in the NCBI Sequence Read Archive (SRA) under accession number GSE291140. The raw proteomic data for early- and late-stage TAMs are available in the iProX repository (https://www.iprox.cn/page/project.html?id=IPX0007435000, accessed on 25 May 2026) under the ProteomeXchange accession PXD046492. The single-cell RNA-sequencing (scRNA-seq) datasets analyzed in this study are available in the NCBI Gene Expression Omnibus (GEO) under accession numbers GSE225063 (melanoma), GSE236581 (colorectal cancer), and GSE183904 (gastric cancer).

## Supplementary Materials

The following supporting information can be downloaded at: https://www.mdpi.com/article/10.3390/ijms27146154/s1.

## Abbreviations

The following abbreviations are used in this manuscript:

TAMs	tumor-associated macrophages
TME	tumor microenvironment
DEGs	differentially expressed genes
LD	Linear dichroism
KEGG	kyoto encyclopedia of genes and genomes
PCA	principal component analysis
DEPs	differentially expressed proteins
GO	gene ontology
BMDMs	bone marrow-derived macrophages
AMPK	AMP-activated protein kinase
MDSCs	myeloid-derived suppressor cells
MDA	malondialdehyde
TCEP	Tris (2-carboxyethyl) phosphine hydrochloride
DTT	Dithiothreitol
2ME	2-Mercaptoethanol
PPP	pentose phosphate pathway
GPX4,	glutathione peroxidase 4
G6PD	glucose-6-phosphate dehydrogenase
LDHB	lactate dehydrogenase B
