# Supplementary material for: AMPK Suppresses Multiple Forms of Cell Death Including Disulfidptosis in Tumor-Associated Macrophages During Tumor Progression
Source: Int J Mol Sci. 2026 Jul 9;27(14):6154. doi: 10.3390/ijms27146154 (PMC13410161; doi:10.3390/ijms27146154)
Supplement: Supplementary file 1 [file ijms-27-06154-s001.zip › ijms-4381826-supplementary/Supplementary Materials/IJMS-proofread version Supplemental Figures.pdf]

AMPK Suppresses Multiple Forms of Cell Death Including Disulfidptosis in Tumor-Associated Macrophages During Tumor Progression

Ruixuan Wang, Huan Wang, Dianyuan Zhao, Wenting Yang, Di Liu * and Li Tang *

State Key Laboratory of Medical Proteomics, National Center for Protein Sciences (Beijing), Academy of Military Medical Sciences, Beijing 102206, China.

* Correspondence: diliu09@ncpsb.org.cn (D.L.); tangli@ncpsb.org.cn (L.T.); Tel./Fax: +86-10-61777087 (D.L.); +86-10-61777091 (L.T.)

Supplementary Figures S1-S4

Figure S1

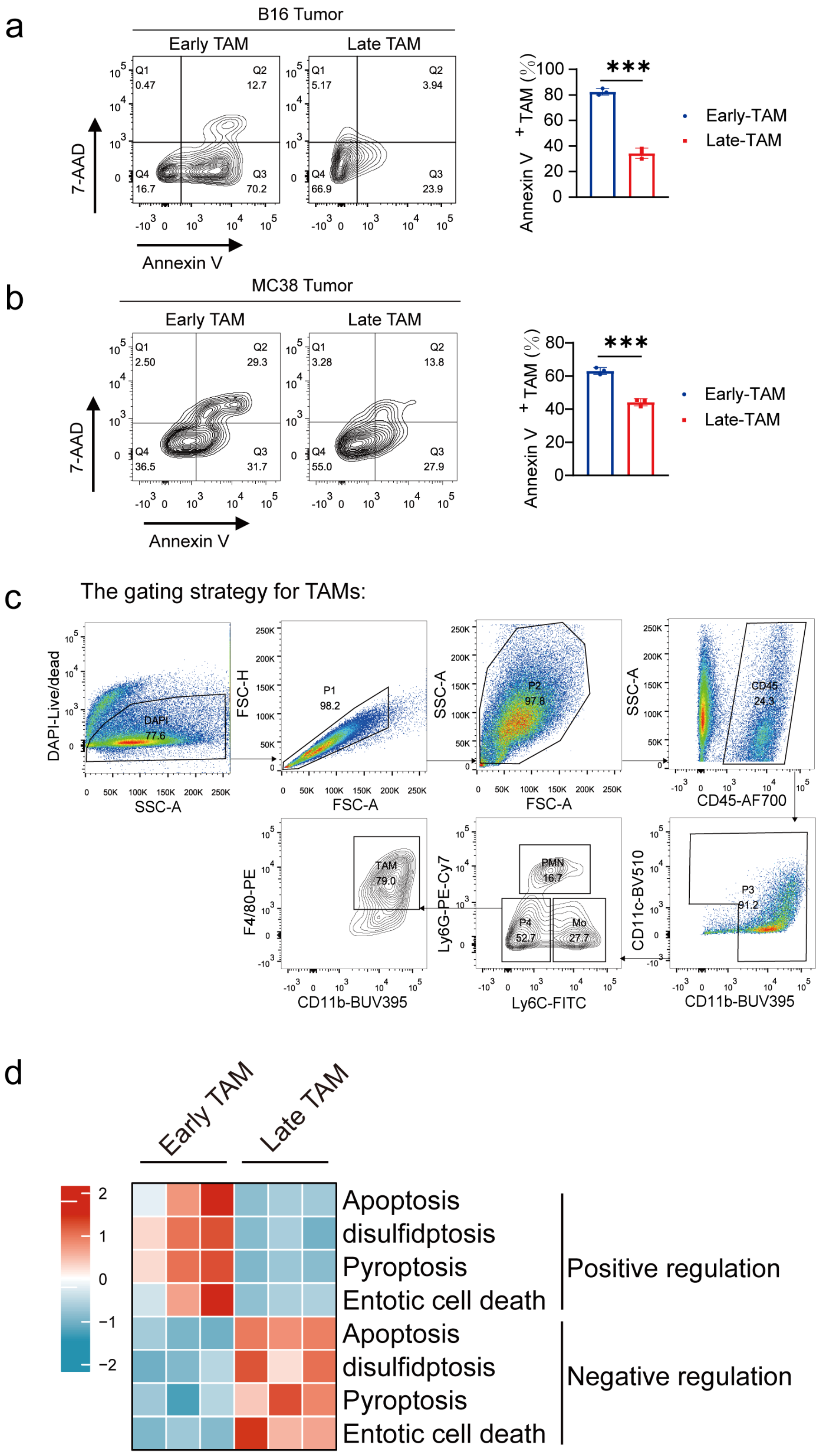

Figure S1. The apoptosis of TAMs is reduced during tumor progression.

(a) Flow cytometric quantification and representative plots of frequency of Annexin V^+ TAMs from B16 tumor-bearing mice in the early (day 8) and late stages (day 15). (b) Flow cytometric quantification and representative plots of frequency of Annexin V^+ TAMs from MC38 tumor-bearing mice in the early (day 8) and late stages (day 15). (c) Gating strategy for TAMs. TAMs were identified as $CD45^+CD11b^+Ly6C^-Ly6G^-F4/80^+$cells

(d) Based on normalized transcriptomic data of early- and late-stage TAMs, gene sets positively and negatively regulating apoptosis, necroptosis, pyroptosis, and disulfidptosis, were scored using the calculate*_sig_score function* in the IOBR package (v0.99.9). The heatmap displays the enrichment scores of these positively and negatively regulated cell death–related gene sets. Data are presented as mean ± SEM. *$p < 0.05$; **$p < 0.01$; ***$p < 0.001$.

Figure S2

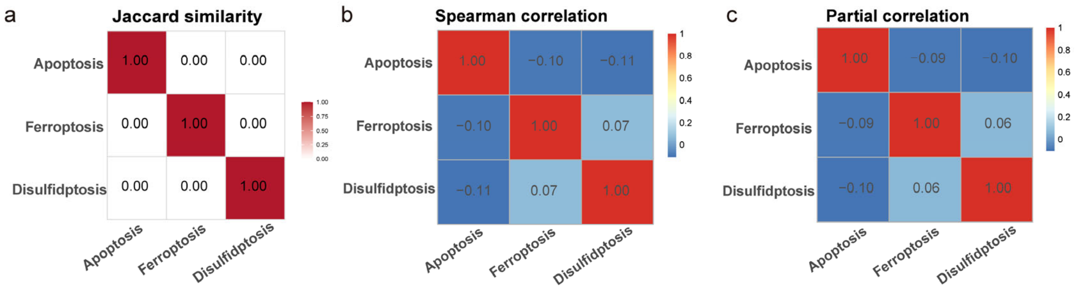

Figure S2. Assessment of gene set overlap and signature independence among apoptosis, ferroptosis, and disulfidptosis programs in melanoma TAMs.

(a) Jaccard similarity analysis of gene set overlap among apoptosis, ferroptosis, and disulfidptosis signatures. Pairwise Jaccard indices were calculated to quantify the degree of gene overlap between the three curated cell death–related gene sets.

(b) Spearman correlation analysis of apoptosis, ferroptosis, and disulfidptosis signature scores across melanoma samples based on TAMs derived from single-cell RNA-seq data. Signature scores were calculated from TAM populations within each sample to evaluate pairwise relationships among the three cell death-related programs.

(c) Partial correlation analysis of the corresponding signature scores across melanoma samples after adjustment for shared variance among signatures.

Figure S3

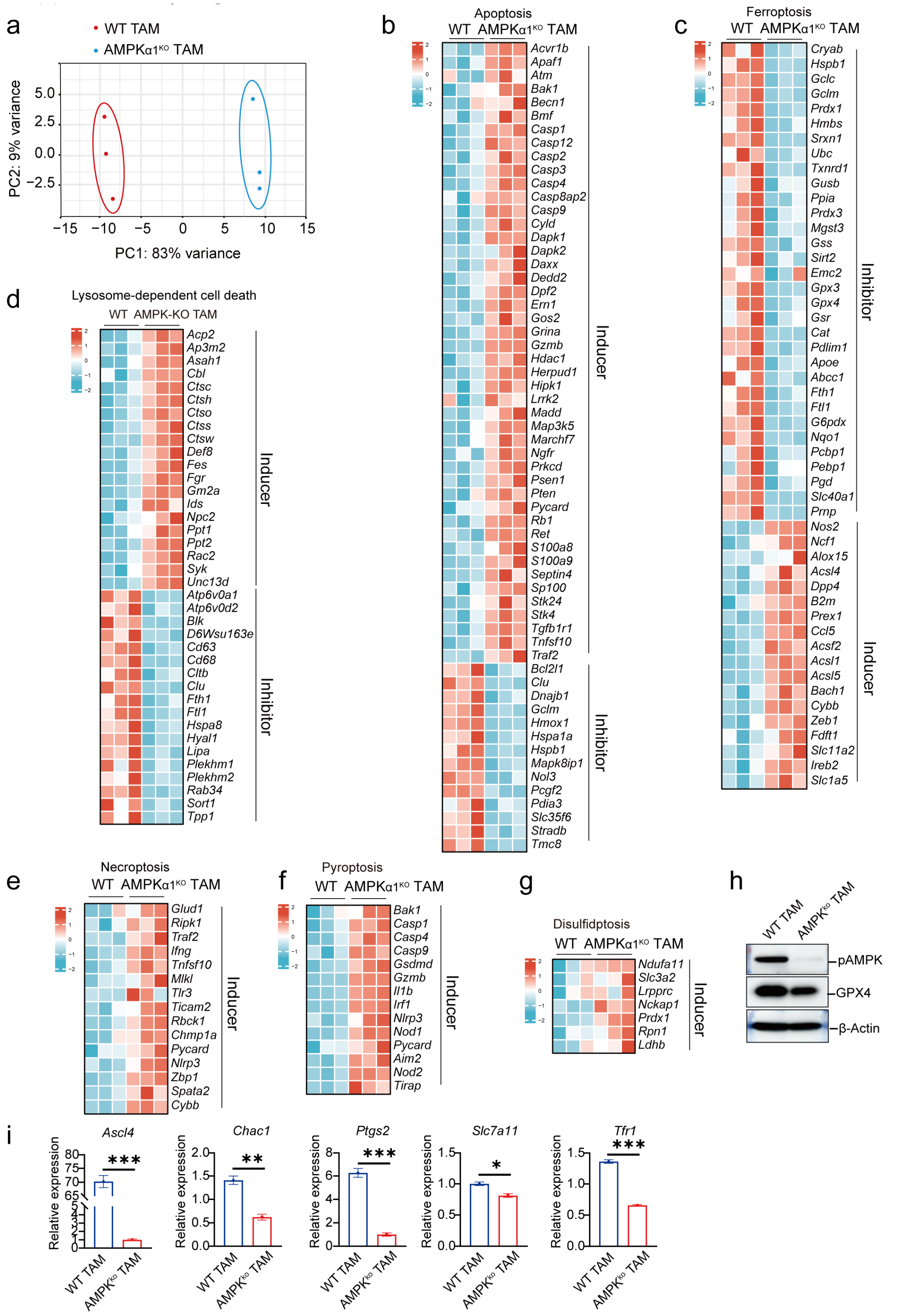

Figure S3. Death-related gene expression profile and activation of multiple

cell death modalities in AMPK^{KO} TAMs

(a) PCA analysis of WT TAM and AMPK^{KO} TAM sorted from the tumor of B16 tumor-bearing mice *AMPK$^{fl/fl}$* (*WT*) and *AMPK$^{fl/fl}$LyZ2^{cre}* (*AMPK-cKO*).

(b-g) Heatmap of promoting (Inducer) and inhibitory (Inhibitor) genes for apoptosis, ferroptosis, necroptosis, pyroptosis, lysosome-dependent cell death and disulfidptosis.

(h) Western blot analysis of GPX4 expression in WT and AMPK^{ko} TAMs induced with B16 CM for 3 days.

(i) The relative mRNA expression of ferroptosis-related genes *Slc7a11*, *Ptgs2*, *Tfr1*, *Ascl4* and *Chac1* in WT and AMPK^{KO} TAMs induced with B16 CM. Data are presented as mean ± SEM. *p < 0.05; **p < 0.01; ***p < 0.001.

Figure S4

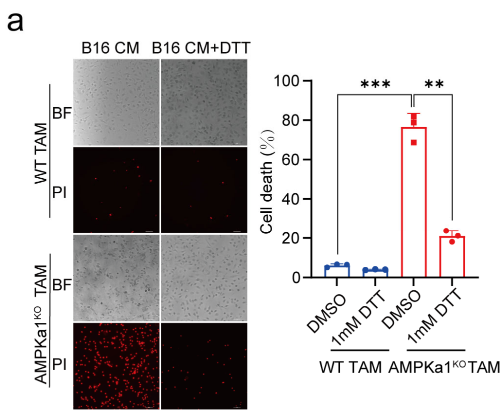

Figure S4. AMPK deficiency-induced TAM death was suppressed by the disulfidptosis inhibitor DTT.

(a) Representative PI staining image and quantitative analysis of death rates of WT and AMPK^{KO} BMDMs stimulated with B16 CM and treatment with disulfide stress inhibitors DTT (1mM) for 108h. Scale bar = 50 μm. TAM death rates were assessed by Image J (v1.53a).

Figure S5

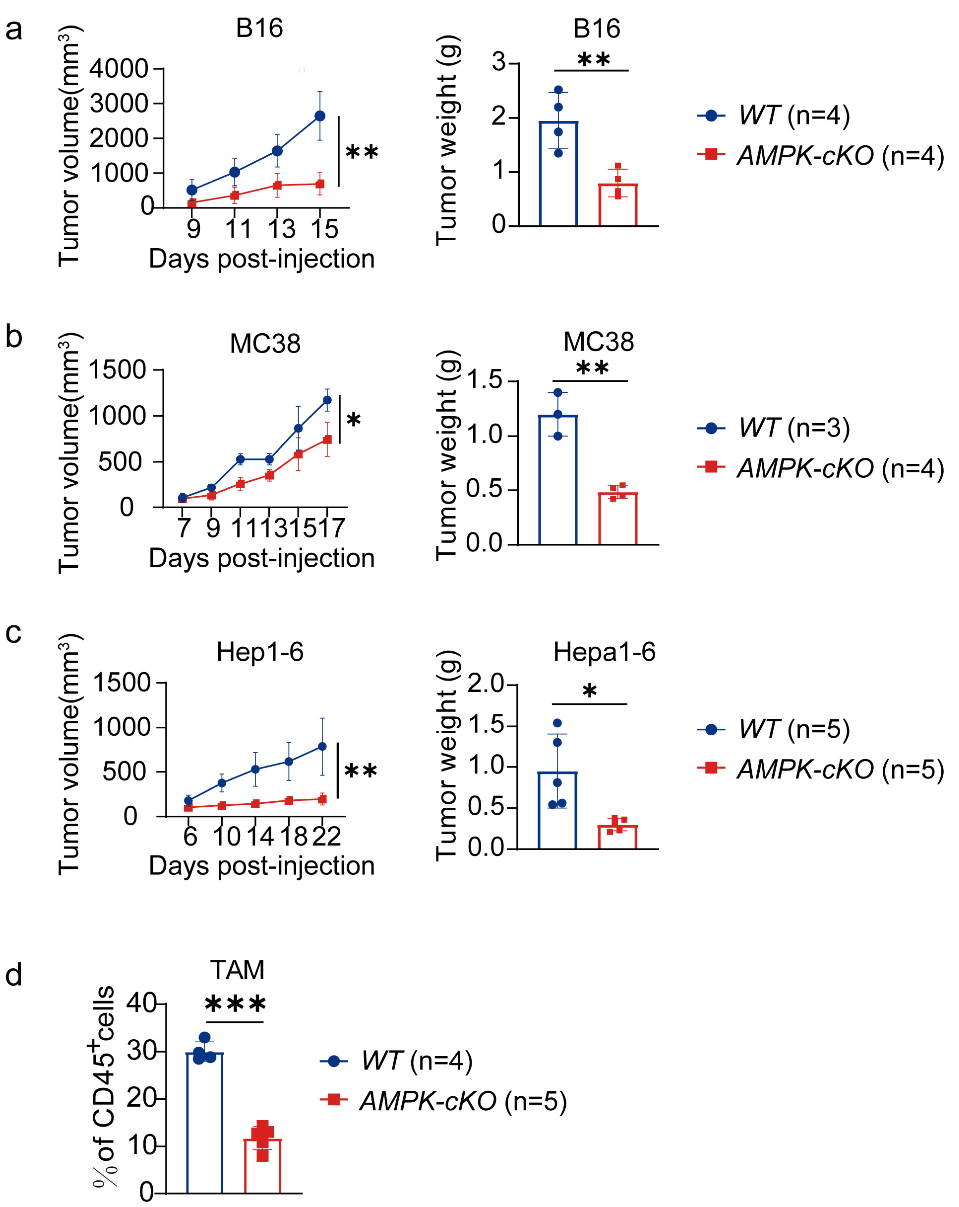

Figure S5. Myeloid AMPK knockout inhibits tumor growth and reduces the proportion of TAMs.

(a) $AMPK^{fl/fl}$ (*WT*) and $AMPK^{fl/fl}LyZ2^{cre}$(*AMPK-cKO*) mice were subcutaneously injected with B16-F10. Tumor volume was measured every three days from the sixth day after tumor cells injection. Tumor weight was measured on the day of sacrifice.

(b) *AMPK*$^{fl/fl}$ (*WT*) and *AMPK*$^{fl/fl}$*LyZ2*^{cre}(*AMPK-cKO*) mice were subcutaneously injected with MC38. Tumor volume was measured every three days from the sixth day after tumor cells injection. Tumor weight was measured on the day of sacrifice.

(c) *AMPK*$^{fl/fl}$ (*WT*) and *AMPK*$^{fl/fl}$*LyZ2*^{cre}(*AMPK-cKO*) mice were subcutaneously injected with Hepa 1-6. Tumor volume was measured every three days from the sixth day after tumor cells injection. Tumor weight was measured on the day of sacrifice.

(d) Flow cytometric quantification of frequency of the TAMs ($CD45^+CD11b^+Ly6G^-Ly6C^-F4/80^+$). Data are presented as mean ± SEM. *$p < 0.05$; **$p < 0.01$; ***$p < 0.001$.
