# Supplementary material for: AMPK Suppresses Multiple Forms of Cell Death Including Disulfidptosis in Tumor-Associated Macrophages During Tumor Progression
Source: Int J Mol Sci. 2026 Jul 9;27(14):6154. doi: 10.3390/ijms27146154 (PMC13410161; doi:10.3390/ijms27146154)
Supplement: Supplementary file 1 [file ijms-27-06154-s001.zip › ijms-4381826-supplementary/Supplementary Materials/Supplementary table S1. List of primer.pdf]

Supplementary table S1. List of primer

The primer of Q-PCR:

**Gene**	**primer**
β-actin	F:TCCATCATGAAGTGTGACGT<br>R:TACTCCTGCTTGCTGATCCAC
Acsl4	F:ATTGGTCAGGGATATGGGCT<br>R:AGAGGAGCTCCAACTCTTCCA
Slc7a11	F:TGGGTGGAACTGCTCGTAAT<br>R:AGGATGTAGCGTCCAAATGC
Ptgs2	F:GGGAGTCTGGAACATTGTGAA<br>R:GTGCACATTGTAAGTAGGTGGACT
Chac1	F: TGACCCTCCTTGAAGACCGTGA<br>R:AGTGTCATAGCCACCAAGCACG
Tfr1	F:GTTTCTGCCAGCCCCTTATTAT<br>R:GCAAGGAAAGGATATGCAGCA

The primer of mouse genotype:

Lyz2cre	3066:CCCAGAAATGCCAGATTACG<br>3067:CTTGGGCTGCCAGAATTTCTC<br>3068:TTACAGTCGGCCAGGCTGAC
AMPKα1-flox	11528： CCCACCATCACTCCATCTCT<br>11529： AGCCTGCTTGGCACACTTAT
